# Detection of potential complications in cancer survivors after chemotherapy and development of a regional care network: the PASCA feasibility study

**DOI:** 10.3389/fmed.2025.1469930

**Published:** 2025-03-04

**Authors:** Romain Buono, Olivia Pérol, Meyssane Djebali, Mélodie Borja, Alicia Abadie, Stéphane Morisset, Anne-Sophie Michallet, Aude Fléchon, Helen Boyle, Emmanuelle Nicolas-Virelizier, Philippe Rey, Yann Guillermin, Souad Assaad, Amine Belhabri, Laure Lebras, Jean-Yves Blay, Béatrice Fervers, Mauricette Michallet

**Affiliations:** ^1^Department of Prevention, Cancer and Environment, Léon Bérard Cancer Center, Lyon, France; ^2^Department of Medical Oncology, Léon Bérard Cancer Center, Lyon, France; ^3^INSERM U1296, Léon Bérard Cancer Center, Lyon, France

**Keywords:** feasibility study, cancer survivors, complication detection, testicular germ cell tumors, hematological malignancy

## Abstract

Complications are often poorly identified and managed in cancer survivors after treatment and restoring their initial quality of life remains a challenge, particularly in a context of unequal access to care nationwide. The PASCA “Parcours de Santé au cours du Cancer [in English: healthcare pathways with cancer]” feasibility study was conducted in the Léon Bérard Comprehensive Cancer Center (Lyon, France) to assess the feasibility of a complications detection program, in cancer survivors who have received intensive chemotherapy. An initial network of physicians and healthcare professionals was also set up to facilitate medical referrals after detection. The study had a high recruitment rate (83.8%) and an adherence rate of 43%. In our analysis population (*n* = 98), 8% presented *de novo* dermatological, cardiological, and pneumological complications. Of these, 42 completed all program visits. Among them, the number of patients who developed a ≥ grade 2 complication increased between the first and last visits in: nephrology (+13.9%), overweight/obesity (+12.5%), endocrinology (+8.3%) and cardiology (+5.6%). Patient satisfaction was high (68%). The results supported the feasibility of a complication detection program and highlighted the presence of *de novo* complications at the first visit, as well as an increase in the number of patients developing complication in four areas between the first and last visit. In the future, after-treatment programs could be improved by increasing the motivation of the referring oncologists and patients, improving communication and by adapting the follow-up visits to take into consideration the constraints and profiles of the cancer survivors.

## 1 Introduction

The number of cancer survivors is growing substantially worldwide (1–3), due to the aging of the population (4), increasing incidence rates, as well as diagnostic and therapeutic advances, leading to improved cancer survival rates (5).

Of all the types of cancer treatment available, chemotherapy is still widely used and remains strongly associated with the development of adverse effects, such as cardiac, pulmonary, endocrine, renal, overweight/obesity and dermatological complications that affect the health and quality of life of cancer survivors (6–11).

The incidence of heart failure (HF) induced by anthracyclines, which are commonly used to treat many hematological malignancies, ranges from 1 to 48% (12). Bleomycin can cause interstitial lung disease with the presence of a restrictive syndrome and also pulmonary fibrosis (6, 13, 14). Patients also appear to have a high rate of endocrine complications, including hypogonadism (65%) and hypothyroidism (9%) (15, 16). Patients receiving cisplatin appear to suffer from long-term deterioration of renal function (20–30%), with a 20 to 30% reduction in the glomerular filtration rate (GFR) (7, 17, 18). Renal toxicity is also observed in patients who received bendamustine, chlorambucil, cyclophosphamide and fludarabine (19). Chemotherapy-induced weight gain with a change in body mass index (BMI) class, is another frequently observed complication in nearly one third of lymphoma patients, 18 months after diagnosis (20). Dermatological toxicity is observed after multiple cytotoxic agents, affecting hair and nails, the skin barrier, and mucous membranes (21).

In France, two national surveys conducted 2 years (VICAN2) and 5 years (VICAN5) after a cancer diagnosis reported that 44% of adult patients had a degraded physical quality of life, and specific management of complications was reported for only 26% of patients presenting one or more complications (22). The French prospective national CANTO cohort study also showed a poor or deteriorated quality of life trajectory in 16.6% of breast cancer survivors treated with chemotherapy over a period of 4 years following diagnosis (23).

The Europe’s Beating Cancer Plan (24) stressed the need to anticipate, personalize and improve the implementation of patient follow-up, the management of complications and the integration of tertiary prevention in the care pathways for adult cancer survivors (25). This requires earlier detection and better management of complications, better coordination and communication between healthcare professionals, the promotion of research programs to improve understanding of the mechanisms of onset of complications, and the most appropriate ways of managing their consequences (5). The decline of medical density and shortage of hospital staff, overcrowding of cancer services, as well as unequal geographical distribution of healthcare professionals may represent a barrier to healthcare access for cancer survivors care and increase existing inequalities (26–28).

In the present study, the Léon Bérard Comprehensive Cancer Center (Lyon, France) assessed the feasibility of systematic screening six complication categories (i.e., cardiac, nephrology, pneumology, overweight/obesity, endocrinology and dermatology) in adult cancer survivors treated for hematological malignancies or testicular germ cell tumor (TGCT), who had finished treatment with at least one line of intensive chemotherapy. The aim of this feasibility study was to assess the ability of our program to detect complications in cancer survivors who had received intensive chemotherapy using a regional care network.

## 2 Methods

The PASCA study was a prospective, single-center, feasibility study conducted from 2018 to 2020 at the Léon Bérard Comprehensive Cancer Center, Lyon, France. The study protocol was approved by the Léon Bérard Comprehensive Cancer Center Review Board and was registered with the National Commission for Data Protection and Liberties (CNIL) (reference number: 2016177 v0). All participants provided written informed consent.

### 2.1 Study population

The study was proposed to eligible patients during a medical surveillance consultation with their oncologist. During this consultation, the oncologist provided a full explanation of the study objectives and the protocol. The eligibility criteria were: (1) aged 18–75 years, (2) treated with at least one intensive chemotherapy line, (3) treated for a TGCT or a hematological malignancy [multiple myeloma (MM), non-Hodgkin lymphoma (NLH), Hodgkin lymphoma (HL) or acute myeloid leukemia (AML)], followed or not by an allogenic (allo-HCT) or autologous stem cell transplantation (ASCT), (4) have achieved complete remission according to the reference standard, (5) have completed at least one medical surveillance visit between May 2018 and December 2020, (6) no history of psychiatric disorders, (7) have given written consent to participate. All patients who completed the first study visit were included in the PASCA study population study.

### 2.2 Detection of post-treatment complications

The complications of interest in the study were selected on the basis of two factors: (1) the absence of systematic screening and management within the center, and (2) the limited number of prospective and longitudinal data in the literature.

Following the oncological or hematological follow-up consultation, screening for cardiac, nephrology, pneumology, overweight/obesity, endocrinology and dermatology complications was carried out. The screening lasted about 90 min and included:

- questionnaires: Medical Research Council (MRC) and Chronic obstructive pulmonary disease Assessment Test™ (CAT) (29, 30), Global HRQoL (Health related quality of life) (EORTC QLQ-C30 v3.0), Fatigue Severity Scale (FSS);
- laboratory tests:
  - blood: creatinine, GFR estimation (CKD-EPI creatinine equation), NT-proBNP, fasting blood glucose, LDL-cholesterol (low-density lipoprotein) (Friedwald equation), triglycerides, thyroid-stimulating hormone (TSH), 25-hydroxyvitamin D, total testosterone;
  - urine: proteinuria, hematuria and leukocyturia;
- medical examination: blood pressure, heart rate, electrocardiogram, weight, height, waist circumference;
- physical test: 6-min walk test (6MWT).

Patients attended a second and a third follow-up visit at 6 and 12 months after the first visit, respectively, when similar clilnical and biological investigations were carried out.

The research staff involved were as follows: (1) the investigating physicians who included the patients, (2) a clinical research associate who supervised the completion of the questionnaires, (3) a clinical research nurse who carried out the biological sampling, (4) a physical activity teacher who supervised the physical testing, (5) the study coordinating physician who carried out the medical examination and assessed the complications.

Before the start of the study, the study coordinator physician created a grading system for each complication (Table 1):

- Grade 0: normal result with no clinical impact related to the studied complication;
- Grade 1: presence of abnormal result(s) related to the studied complication, with an uncertain clinical impact;
- Grade 2: one or more abnormal result(s) related to the studied complication with a clinical impact, and with a previous history prior to the cancer diagnosis;
- Complication: one or more abnormal result(s) related to the studied complication with clinical impact, and with no history prior to the cancer diagnosis, corresponding to a *de novo* complication.

**Table 1:** Variables used to determine the grading of detected complications.

Complication category	Variables included in the grading
Cardiology	Systolic and diastolic blood pressure, ECG parameters (sinus rhythm appreciation, heart rate, QRS axis, duration of P wave, QRS, QT corrected and PR space, appearance of P, Q, T waves and QRS’s appearance/amplitude in precordial leads), LDL-c (Friedwald formula), triglycerides and NT-proBNP plasma concentration, data from patient record on cardiological history before the diagnosis of cancer, current cardiological follow-up and on history of cardiotoxic treatments: anthracyclines, thoracic/mediastinal irradiation, alkylating agents (cyclophosphamide) and proteasome inhibitors (carfilzomib, bortezomib)
Nephrology	Creatininemia, GFR (CKD-EPI formula) and its variation from a previous value, proteinuria, hematuria and leucocyturia by urine dipstick and read by the Siemens Clinitek Status+® analyzer, data from patient record on nephrological history before the diagnosis of cancer
Pneumology	Data from dyspnea MRC and CAT questionnaires and from patient record on pneumological history before the diagnosis of cancer
Overweight/obesity	BMI and waist circumference and data from patient record on BMI values before the diagnosis of cancer
Endocrinology	Fasting blood glucose, total testosterone, TSH, 25-hydroxyvitamin D, data from patient record on endocrinological history before the diagnosis of cancer
Dermatology	Maculo papular exanthema, photosensitivity rash, dry skin, hand-foot syndrome, data from patient record on dermatological history before the diagnosis of cancer

The following cut-off values of the French High Authority for Health (HAS) or French or European learned societies were used by the study coordinator physician to detect any abnormal results: cardiology (8, 12, 31), nephrology (32), pneumology (29, 30, 33), overweight/obesity (34), endocrinology (35–37), and dermatology (38). When several guidelines were available, selection was based on the following criteria: availability and applicability of the guideline to the cancer survivor population. When no guidelines were available for cancer survivors, the cut-offs available for the general population were applied. The questionnaires were interpreted in accordance with the recommendations of the authors (30–33).

For a given study visit and for a given complication, if two different grades were identified in a patient, the higher grade was systematically retained. Example of a fictitious patient’s cardiological assessment at his first study visit: hypertension measured with an average of 150/90 mmHg and no known history of hypertension at the time of diagnosis would generate a grade of “Complication.” If he also had dyslipidemia of 1.8 g/L chronically treated with lipid-lowering drugs at the time of diagnosis, it would generate a “Grade 2.” His highest cardiological grade would be “Complication,” and he would therefore be classified in this group for this visit.

The questionnaires were interpreted in accordance with the recommendations of the authors (30–33).

After each visit and for each complication category, the study coordinator physician applied his grading system, and based on the clinical impact for the patient, decided to either provide an oral recommendation to the patient by telephone, refer the patient to their general practitioner or refer them to another healthcare professional.

### 2.3 Patients’ satisfaction

Patient satisfaction was assessed during the third follow-up visit, using a 4-points Likert scale on four items self-completed by the patients: (1) usefulness of the PASCA program, (2) quality of the information provided by the study team, (3) quality of the orientation provided after the visits, and (4) satisfaction about the length of each visit. The response items were “totally disagree/agree a little bit/agreed somewhat/totally agree” for scales (1), (2), and (3); and “little long/acceptable length/short length/very short length” for scale (4).

### 2.4 Regional care network

We set up a regional network of healthcare professionals specifically for the study, throughout the Auvergne-Rhône-Alpes (AURA) region (8.1 million inhabitants) in order to refer patients, when needed, to a healthcare professional as close as possible to their home and to reduce referral problems. About 45% of the patients attending the Léon Bérard Comprehensive Cancer Center live in Metropolitan Lyon and the Rhône Department (1.9 million inhabitants), and 55% live in neighboring departments (4.5 million inhabitants) or farther away.

For each new patient entering the program, a request to affiliate with the PASCA network was sent by mail or e-mail (with a telephone reminder in case of no response) to the patient’s general practitioner, explaining the objectives of the program and their role in an information leaflet. Positive responses to the request to affiliate were considered as acceptance, and negative responses and non-responses as a refusal.

### 2.5 Outcome measures

The primary endpoints of the study were the recruitment rate (number of patients who completed their first visit/number of patients eligible for the study) and the adherence (number of patients who completed all visits/number of patients who completed their first visit). The secondary endpoints were the patient satisfaction and the percentage of *de novo* complications detected.

### 2.6 Data collection

Three data extractions were performed by a research technician from:

1. data from clinical and tumor characteristics, treatment, medical consultation reports with the onco-hematologist, clinical examinations, history of comorbidities related to the complications of interest before cancer diagnosis were extracted from the patient’s file;
2. data on biological parameters, questionnaires, measurements and physical tests were extracted from the three study visits;
3. the responses to the network affiliation requests received.

### 2.7 Statistical analysis

As the primary outcome of the PASCA study was feasibility, no formal sample size calculation was performed. The sample size in the present study was pragmatic, based on expected recruitment, considering that 90 patients were sufficient to assess the feasibility of the screening procedure. The sample size aligns with published recommendations for feasibility studies (39–42).

Participants’ characteristics were described using median and interquartile range for quantitative data concerning the duration between study timeline (diagnosis, last cancer treatment, first, second and third program visit). Means and standard deviations (SDs) were described for other quantitative data. Frequencies and percentages were used for qualitative data.

Comparative analyses were performed for percentages using Chi-squared or Fisher’s exact tests, and for means using t tests, F-tests or Wilcoxon tests, as appropriate. A type 1 error risk α of 0.05 was used. For multiple comparisons, a Bonferroni correction was applied. A two-sided *p* value < 0.05 was considered as significant.

A linear mixed model for repeated measures was used to evaluate the relationship of the follow-up (visit number) on HRQoL score, fatigue FSS score and 6 minute walk distance (6MWD). The visit number was considered as a continuous variable. The following assumptions were evaluated: relationship between residuals and distribution of residuals. The best fitted model was selected based on likelihood ratio test. Sankey plots were constructed to graphically represent the proportions of patients graded for complications at each of the three visits, as well as the flow of patients changing grades between visits. For each complication, patients with incomplete data availability at all three visits were excluded from the analyses.

Statistical analyses were performed using the tidyverse, lmerTest and the MuMIn package in R software version 4.1.2.

## 3 Results

### 3.1 Patients’ characteristics

**Figure 1:**
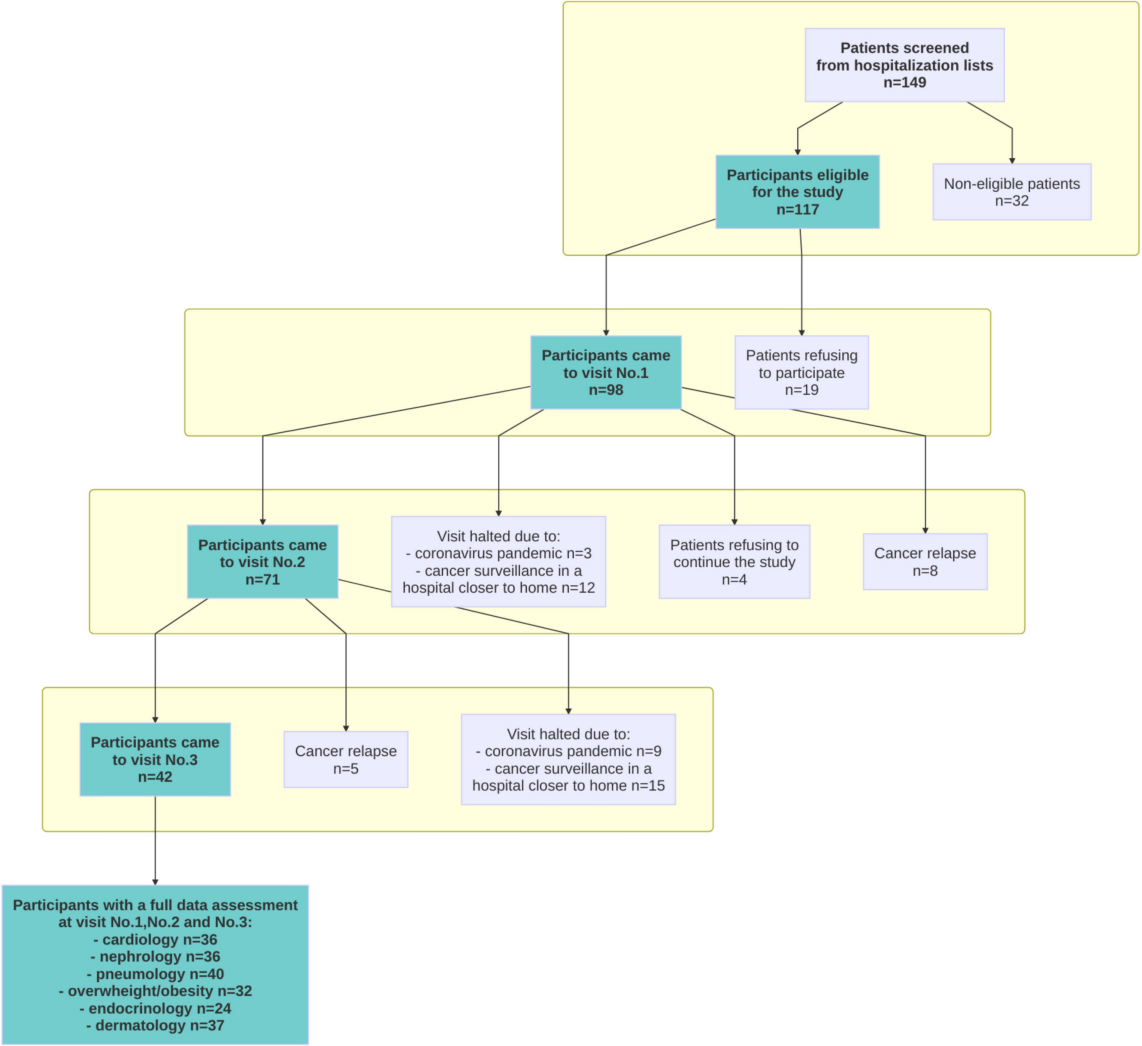
Flowchart of the study.

The baseline demographic and clinical characteristics of the participants are summarized in Table 2. From the 149 patients screened for eligibility, a total of 117 participants were eligible for the present study (Figure 1).

**Table 2:** Demographic and clinical characteristics of the participants.

Characteristic	At least first visit completed *N* = 98 (analysis population)	All three visits completed, *N* = 42	*p*-value
Age (years), mean (SD)	52 (13)	52 (14)	0.97
Sex, *n* (%)			0.52
Male	72 (73)	33 (79)	
Female	26 (27)	9 (21)	
Pathology, *n* (%)			0.74
AML & MDS	8 (8.2)	2 (4.8)	
CLL	2 (2.0)	1 (2.4)	
NHL	28 (29)	8 (19)	
HL	9 (9.2)	3 (7.1)	
MM	29 (30)	16 (38)	
TGCT	22 (22)	12 (29)	
Stem cell transplant, *n* (%)			0.93
allo-HCT	12 (12)	4 (9.5)	
ASCT	51 (52)	22 (52)	
No graft	35 (36)	16 (38)	
Surgery, *n* (%)	25 (26)	14 (33)	0.34
Cancer treatment, *n* (%)			0.74
CT	79 (81)	36 (86)	
CT + RT	18 (18)	6 (14)	
CT + RT + HT	1 (1.0)	0 (0)	
BMI (kg/m^2^), mean (SD)	25.1 (4.6)	25.0 (4.3)	0.86
Cardiac history, *n* (%)	19 (21)	11 (28)	0.42
Pulmonary history, *n* (%)	7 (7.8)	5 (12)	0.51
Kidney history, *n* (%)	4 (4.4)	2 (5.0)	>0.99
Endocrinological history, *n* (%)	6 (6.7)	2 (5.0)	>0.99
Dermatological history, *n* (%)	2 (2.2)	1 (2.5)	>0.99
Lines of treatment, *n* (%)			0.48
First line	74 (76)	34 (81)	
Second line	24 (24)	8 (19)	
Second cancer, *n* (%)	4 (4.1)	1 (2.4)	>0.99
Previous cancer relapse, *n* (%)	21 (21)	7 (17)	0.52

allo-HCT, allogenic hematopoietic cell transplantation; AML, acute myeloid leukemia; ASCT, autologous stem cell transplantation; BMI, body mass index; CLL, chronic lymphocytic leukemia; CT, chemotherapy; HL, Hodgkin lymphoma; HT, hormonotherapy; MDS, myelodysplastic syndrome; MM, multiple myeloma; NHL, non-Hodgkin lymphoma; RT, radiotherapy; TGCT, testicular germ cell tumor.

A total of 98 patients consented to participate in the study and completed the first program visit these patients were included in the PASCA study analysis population.

Their mean age (±SD) was 52 (±13) years, with a majority of men (73%), and the mean BMI was 25.1 (±4.6). Overall, 77.6% had hematological malignancies and 22.4% had TGCTs. At inclusion, 74 patients had a first line of anticancer treatment and 24 a second line. The cancer treated was a first relapse or a second cancer for 21 and 4.1% of the patients, respectively. Previously 26% of the patients had undergone surgery, 81% had received only chemotherapy and 19% had received chemotherapy combined with radiotherapy or hormonal therapy. Allo-HCT and ASCT were performed in 12 and 52% of patients, respectively. The medical history at diagnosis of their last cancer included: cardiac (21%), pulmonary (7.8%), endocrine (6.7%), renal (4.4%), and dermatological (2.2%) comorbidities.

### 3.2 Recruitment rate

The recruitment rate was 83.8%, i.e., a total of 98 patients consented to participate out of the 117 patients who were identified electronically by the research team, prior to the medical consultation.

### 3.3 Adherence rate

A total of 42 patients of the initial 98 patients attended the three scheduled study visits, giving an adherence rate of 43%. This subpopulation with longitudinal data was representative of the PASCA study analysis population as shown in Table 2. The reasons for not attending all three visits were: change to a peripheral center closer to the patient’s home, either on the initiative of the oncologist or of the patient for 27 patients (28%); appointment canceled because of the SARS-CoV-2 (COVID-19) pandemic for 12 patients (12%); relapse of multiple myelomas for 13 patients (13%); and consent withdrawal after the second visit for 4 patients (4%).

### 3.4 Detection of treatment complications after treatment

The percentage of patients with grade ≥ 2 complications (grade 2 or *de novo* complication) was: 27.6% (overweight/obesity), 13.1% (dermatology), 11.6% (pneumology), 10.2% (cardiology), 5.1% (endocrinology), 2.2% (nephrology). More than 8% of the analysis population (*n* = 98) with a *de novo* complication had dermatology, cardiology, and pneumology complications (Supplementary Table S1). No statistical differences were found between patients with TGCTs and hematological malignancies.

**Figure 2:**
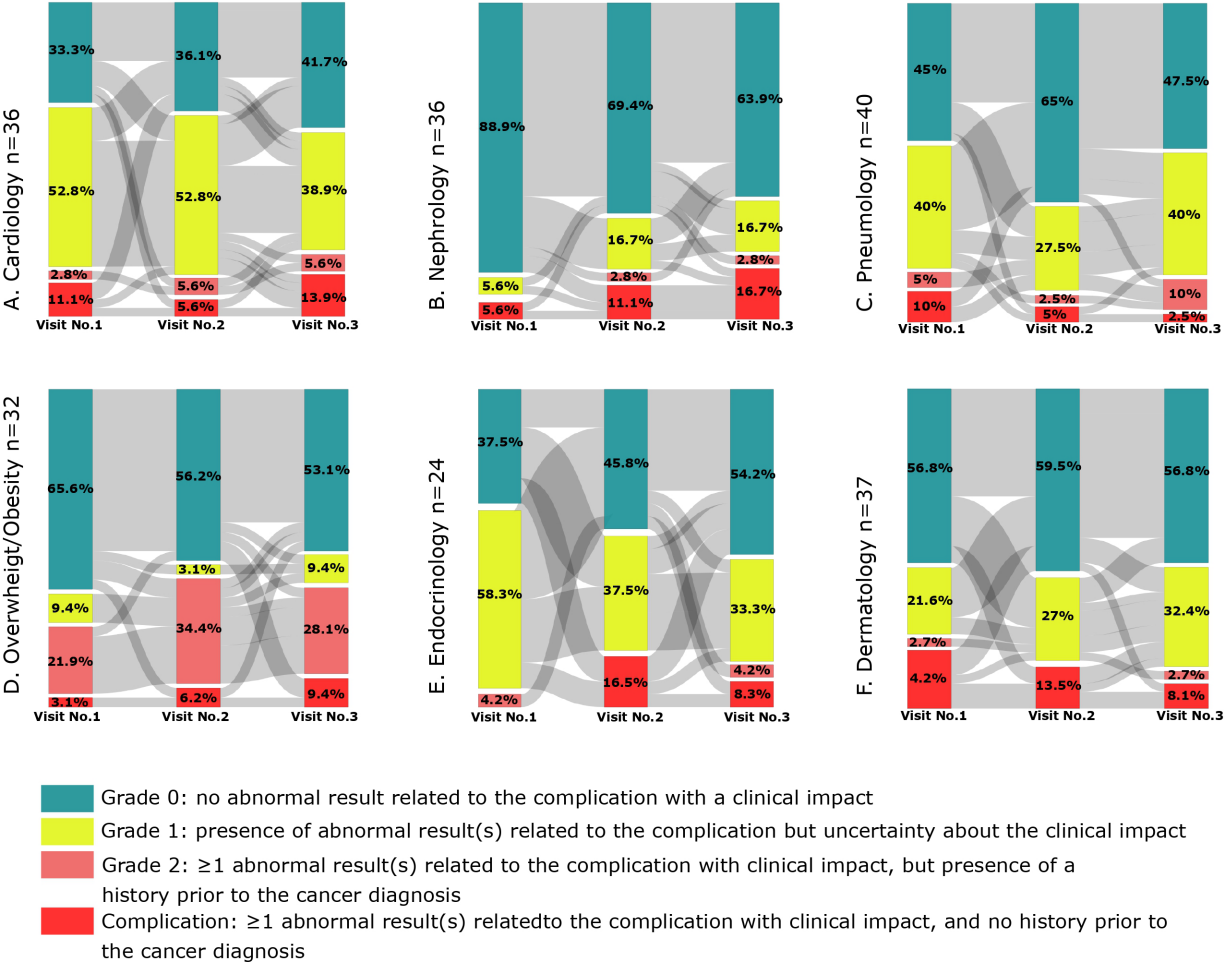
Evolution of grades by visit and by complication.

There was an increase between the first and third visits in the number of patients who developed a grade ≥ 2 complication (grade 2 or de novo complication) among the 42 patients who completed all three study visits (Figure 2). These complications concerned nephrology (+13.9%), overweight/obesity (+12.5%), endocrinology (+8.3%) and cardiology (5.6%). In contrast, we observed a decrease in dermatology (−10.8) and pneumology (−2.5%) complications between the first and last visits. For each category, the differences between visits were not statistically significant. The evolution observed for complication grades between visits and details of patient flow are available in the Supplementary material.

No relationship was found between the overall HRQoL score and the follow-up (number of visits) (*p* = 0.11) (Supplementary Figure S1A). We observed a significant (*p* = 0.003) and negative relationship between the fatigue FSS score and the follow-up (low fatigue FSS score = low intensity fatigue symptoms) (Supplementary Figure S1B), and a significant (*p* = 0.021) and positive relationship between the 6MWT and the follow-up (high 6MWD = high functional capacity) (Supplementary Figure S1C). For both significant relationships, the conditional R-squared was high (54.6 and 68.7%), while the marginal R-squared (4.5 and 2.1%) was very low, respectively.

### 3.5 Patients’ satisfaction

Among the 42 patients who attended the three study visits, 34 (81%) completed the satisfaction questionnaire, 23 (68%) patients “totally agreed,” and 10 (30%) “agreed somewhat” with the statement “the PASCA program is helpful to patients”; 19 (56%) “totally agreed” and 14 (42%) “agreed somewhat” with the statement “the information provided by the healthcare staff is clear and understandable.” Finally, for the question “how do you find the length of each PASCA program visit?,” 24 (60%) responded “acceptable length” and 10 (30%) responded “short length.”

### 3.6 Regional care network

Overall, 120 healthcare professionals out of the 150 contacted agreed to join the network, including 97 physicians (54.6% general practitioners and 45.4% specialists), corresponding to a response rate of 80%. However, an average of two telephone reminders were required to achieve this recruitment level. All the healthcare professionals who agreed to join the network, practiced in the Lyon agglomeration.

## 4 Discussion

To the best of our knowledge, this is the first study assessing the feasibility of systematic screening cancer survivors for complications. We demonstrate the feasibility of a complication screening program in adult cancer patients treated with intensive chemotherapy, with a high recruitment rate and moderate adherence with almost half of participants attending the final visit. Study visits were canceled for 12 patients due to the COVID-19 pandemic and for 27 patients due to a change of hospital center closer to the patients’ homes.

Complications were identified at different visits, with similar rates for patients with TGCT and hematological malignancies. No obvious trends were observed for the number of complications identified between the first and last visits, for the subpopulation of patients who attended all three study visits.

Between the first and third visits, patients showed a significant improvement in their physical condition, with a slight increase in physical testing capacity and a slight decrease in fatigue. For these two significant relationships, high conditional R-squared associated with low marginal R-squared indicate that random effects play a crucial role in the explanatory power of the models. The HRQoL of cancer survivors was lower than that of the general population (weighted for sex and age at inclusion at the first visit) (43), but improved at subsequent visits to be above the average found for the general population, but the change over time (from Visit No.1 to Visit No.3) was not statistically significant.

We tested the program on patients who survived TGCT and hematological malignancies, but it is likely that the tools and the frequency of the visits will need to be adapted depending on the patient profiles. Based on our experience, two patient profiles in particular will require the integration of dedicated tools for complications:

1. chronic graft-versus-host disease, that occurs between 3 and 14 months after allo-HCT in approximately 20% of matched sibling transplantations and 40% of matched unrelated transplantations (44–47);
2. complications induced by the therapy during the induction period for MM patients but which could also been related to the disease itself (48).

Patients with AML who had been treated by allo-HCT were included in our series an average of 142.18 months (SD = 121.39) after diagnosis. They were included mainly to test if it was feasible to include patients in the program irrespective of the delay after the end of the treatment. However, the priority of our program is to detect complications as early as possible after the end of the treatment in order to propose the best possible management and avoid sequelae. In patients who have had allo-HCT it seems important to enroll them on the program earlier, between the third and the sixth months after allo-HCT, in order to allow the acute graft-versus-host disease phase to be managed, but at the same time to take early action on complications.

The study coordinator physician reported that the COPD and CAT questionnaires did not seem to be sufficiently effective for the screening for pulmonary complications in cancer survivors (49). The coordinator thought that the patients’ responses were not very specific, and that objective data would be necessary. Thus a spirometry test and the evaluation of the diffusing capacity of the lung for carbon monoxide could be added to improve screening and to provide a better understanding of the origin of any dyspnea, in particular in the context of obstructive or restrictive syndromes.

Patient satisfaction for the four items evaluated was very good. Their responses indicated that a 90-min visit was the longest they could accept.

We created an initial network of physicians and healthcare professionals specifically for our study. However, the medical specialists recruited were mainly based in the Lyon agglomeration and, therefore, the network did not provide effective coverage of the AURA region, particularly in low-density medical districts, identified using the National Council of the Order of Physicians database. This highlighted the disparities in medical resources in the large AURA region which should be considered before launching this type of program. In France many approaches to address this issue of medical resources are being discussed at the government level, but it will not be easy to implement the solution (26). We think it would be useful to evaluate the advantages of including an expert nurse or doctor in the program to improve it. This professional could prepare a report about the screening visits and call patients to reassure them, explain the results and the preventive health behaviors that could be implemented, and also, to facilitate medical referral.

The results of our feasibility study showed that previously unidentified complications during oncological follow-up could be detected and highlighted what seemed to work well in our complications screening program after cancer treatment and what could be improved. The study provided preliminary longitudinal data on patient complications, HRQoL, fatigue, physical condition, and satisfaction in one French region.

The number of patients who left the program early could have been reduced in two ways. Firstly, for patients whose cancer surveillance was delegated to a local hospital, we could make the oncologist aware of the benefits of the complication screening program and make the patient aware of the importance of the program in preventing sequelae, while at the same time reassuring them. Secondly, for patients whose consent was withdrawn between follow-up visits, we could improve the organization of follow-up visits by taking into consideration the patients’ time constraints, as they gradually return to a normal life during this post-treatment period.

One of the limitations of our study was that the analysis population was small, which is a common limitation in feasibility studies. In addition, although, the study was undertaken during the COVID-19 pandemic, which led to appointments being canceled, it will be important to find solutions to reduce the number of patients leaving the screening program before the last visit. We were able to describe trends over each of the three visits but we could not identify specific profiles of cancer survivors. For the future, we believe that it will be crucial to assess the program in a larger-scale prospective cohort study, including patients with different profiles, pathologies, and treatments patient to enable more comprehensive study of complications arising after cancer treatment.

## 5 Conclusion

The results from the PASCA feasibility study demonstrated the feasibility of a complication screening program in adult cancer survivors involving three visits scheduled after intensive treatment, with a high rate of patient satisfaction. An initial regional network of healthcare professionals was established to facilitate medical referral, and our results highlighted the presence of *de novo* complications at the first visit, as well as an increase in the number of patients developing complication in four categories of complications between the first and third visit. Increasing communication with referring oncologists and patients and motivating them to participate, and a more flexible scheduling of follow-up visits could reduce the number of patients leaving the program before the third visit. It would be interesting to adapt the tools and frequency of visits to the patient’s profile, and to integrate telephone calls to patients after each visit to help them with referrals for any complications detected. A larger cohort study to assess a complications screening program that has been modified to take into consideration these preliminary results and practical feedback has been initiated and results are expected in June 2028.

## Trial registration

Additional information analyzed from patient records was blinded and strictly controlled to respect the patients’ privacy rights. Approval for data review and analyses was granted by the Léon Bérard Cancer Center Review Board.

## Data Availability

The raw data supporting the conclusions of this article will be made available by the authors, without undue reservation.

## Glossary

6MWD: 6 minute walk distance
6MWT: 6 minute walk test
allo-HCT: Allogeneic hematopoietic stem cell transplantation
AML: Acute myeloid leukemia
ASCT: Autologous stem cell transplantation
AURA: Auvergne-Rhône-Alpes
BMI: Body mass index
CAT: COPD Assessment Test^™^
CLL: Chronic lymphocytic leukemia
COVID-19: SARS-CoV-2
CNIL: French National Commission for Data Protection and Liberties
CT: Chemotherapy
FSS: Fatigue severity scale
GFR: Glomerular filtration rate
HAS: French High Authority for Health
HF: Heart failure
HL: Hodgkin lymphoma
HRQoL: Health related quality of life
HT: Hormonotherapy
LDL: Low-density lipoprotein
MM: Multiple myeloma
MRC: Medical Research Council
NHL: non-Hodgkin lymphoma
PASCA: Parcours de Santé au cours du Cancer
RT: Radiotherapy
SD: Standard deviation
TGCT: Testicular germ cell cancer
TSH: Thyroid-stimulating hormone
