# Supplementary material for: Detection of potential complications in cancer survivors after chemotherapy and development of a regional care network: the PASCA feasibility study
Source: Front Med (Lausanne). 2025 Mar 4;12:1469930. doi: 10.3389/fmed.2025.1469930 (PMC11914117; doi:10.3389/fmed.2025.1469930)

Supplementary materials

Detection of potential complications in cancer survivors after chemotherapy and development of a local care network: the PASCA feasibility study

*Descriptive analysis of cardiac complications*

Among the 36 patients with complete cardiac assessment at all three visits (Figure 2.a), 4 (11.1%) patients were already assigned to the "complication" category at the first visit. Reasons for this classification were as follow: major high blood pressure (n=2); history of known cardiotoxic treatment (doxorubicin and thoracic radiotherapy) (n=2) never monitored by a cardiological examination (n=1); and hyper LDL-cholesterolemia (n=1). At the second visit, 2 (5.6%) patients overall were assigned to the "complication" category, of which one patient had the same classification as at the first visit and one patient was previously classified as “Grade 0” at first visit. The reasons for these classifications were: a history of known cardiotoxic treatment (doxorubicin and thoracic radiotherapy) (n=1); never monitored by a cardiological examination (n=1); an elevation above the norms of NT-proBNP (n=1). At the third visit, 13.9% (n=5) patients were classified in the “complication” category. Two patients had a prior complication grading at the first and/or second visit. The reasons for these classifications were: a history of known cardiotoxic treatment (doxorubicin and thoracic radiotherapy) (n=2); cardiological surveillance discontinued by the patient (n=2); a non-sinusal rhythm (n=1); a high blood pressure (n=1); a significant fasting blood glucose level (n=1).

*Descriptive analysis of nephrology complications*

Among patients with a complete nephrological data (Figure 2.a) assessment at all three visits (n=36), 5.6% (n=2) were already assigned to the "Complication" category at the first visit. The reason(s) for this classification was/were as follows: a significative hematuria (n=1), major high blood pressure with a suboptimal eGFR.

At the second visit, 11.1% (n=4) patients were assigned to the "Complication" category, of which one patient had the same classification as at the first visit, two patients were previously classified as “Grade 0” and one patient as “Grade 1” at first visit. The reason(s) for these classifications were: a significative hematuria (n=1), a suboptimal eGFR (n=1), a decrease > 20% between the two eGFR results of the first and the second visit (n=2).

At the third visit, 16.7% (n=6) patients were classified as “complication”. Four patients had a prior complication grading at the first and/or second visit. The reason(s) for these classifications were: a significative proteinuria (n=2), a suboptimal eGFR (n=4).

*Descriptive analysis of pneumology complications*

Among patients with a complete pneumological data (Figure 2.a) assessment at all three visits (n=40), 10% (n=4) were already assigned to the "Complication" category at the first visit. The reason(s) for this classification was a dyspnea MRC scale ≥ 2 (n=4).

At the second visit, 5% (n=2) patients were assigned to the "Complication" category, one patient was previously classified as “Grade 0” and the other as “Grade 1” at first visit. The reason(s) for these classifications were: a dyspnea MRC scale ≥ 2 (n=1) and a CAT scale with a high impact (n=1).

At the third visit, 2.5% (n=1) patient was classified as “complication” and had a prior complication grading at the second visit. The reason(s) for these classifications was a dyspnea MRC scale ≥ 2.

*Descriptive analysis of overweight/obesity complications*

Among patients with a complete nephrological data (Figure 2.a) assessment at all three visits (n=32), 3.2% (n=1) was already assigned to the "Complication" category at the first visit. The reason for this classification was an overweight with a BMI of 27.5 with an increase of +2.5 point compared to diagnosis.

At the second visit, 6.2% (n=2) patients were assigned to the "Complication" category, of which one patient had the same classification as at the first visit, the other patient was previously classified as “Grade 0”. The reason(s) for these classifications was an overweight with a BMI of 26.6 and 27 (+1.6 point and -0.5 point compared to the first visit, respectively).

At the third visit, 9.4% (n=3) patients were classified as “complication”. One patient had a prior complication grading at the first and the second visit, two patients were previously classified as “Grade 0”. The reasons for these classifications were: a significative proteinuria (n=2), a suboptimal eGFR(n=4). The reason(s) for these classifications was an overweight with a BMI from 25.3 to 28.1. The only patient with a complication status at each visit presented an increase of +1.1 point compared to the second visit.

*Descriptive analysis of endocrinology complications*

Among patients with a complete endocrinological data (Figure 2.a) assessment at all three visits (n=24), no patients were assigned to the "Complication" category at the first visit.

At the second visit, 16.5% (n=4) patients were assigned to the "Complication" category, of which one patient had the same classification as at the first visit, two patients were previously classified as “Grade 0” and two others as “Grade 1” at first visit. The reason(s) for these classifications were: a suspicion of primary hyperthyroidism (n=1), a suspicion of hypogonadism (n=2), a 25-hydroxyvitamin D deficiency (n=1).

At the third visit, 8.3% (n=2) patients were classified as “complication”. One patient had a prior complication grading and the other was previously classified as “Grade 0” at the second visit. The reason(s) for these classifications were: a suspicion of hypogonadism (n=1), a 25-hydroxyvitamin D deficiency (n=1).

*Descriptive analysis of dermatology complications*

Among patients with a complete dermatological data (Figure 2.a) assessment at all three visits (n=37), 18.9% (n=7) were already assigned to the "Complication" category at the first visit. The reason(s) for this classification was/were as follows: a dry skin (n=6), a hand-foot syndrome (n=1).

At the second visit, 13.5% (n=5) patients were assigned to the "Complication" category, of which three patients had the same classification as at the first visit, and two patients were previously classified as “Grade 0” at first visit. The reason(s) for these classifications were: a dry skin (n=4), an exanthem (n=1).

At the third visit, 8.1% (n=3) patients were classified as “complication”. Two patients had a prior complication grading at the second visit. The reason for these classifications was a dry skin (n=4).

Supplementary Table 1. Description of complications per population and per study visit.

|  | Analysis population  N = 98 | | | Hematologic  malignancies  N = 76 | | | Testicular Germ  Cell Cancer  N = 22 | | |
| --- | --- | --- | --- | --- | --- | --- | --- | --- | --- |
|  | Visit No.1 | Visit No.2 | Visit No.3 | Visit No.1 | Visit No.2 | Visit No.3 | Visit No.1 | Visit No.2 | Visit No.3 |
| Cardiology grade, n (%) | N=88 | N=63 | N=41 | N=71 | N=46 | N=30 | N=17 | N=17 | N=11 |
| *0* | 36 (41) | 24 (38) | 17 (41) | 25 (35) | 14 (30) | 13 (43) | 11 (65) | 10 (59) | 4 (36) |
| *1* | 43 (49) | 33 (52) | 15 (37) | 38 (54) | 28 (61) | 9 (30) | 5 (29) | 5 (29) | 6 (55) |
| *2* | 1 (1.1) | 2 (3.2) | 2 (4.9) | 1 (1.4) | 2 (4.3) | 2 (6.7) | 0 (0) | 0 (0) | 0 (0) |
| *Complication* | 8 (9.1) | 4 (6.3) | 7 (17) | 7 (9.9) | 2 (4.3) | 6 (20) | 1 (5.9) | 2 (12) | 1 (9.1) |
| Nephrology grade, n (%) | N=91 | N=66 | N=41 | N=70 | N=48 | N=30 | N=21 | N=18 | N=11 |
| *0* | 82 (90) | 48 (73) | 26 (63) | 65 (93) | 39 (81) | 17 (57) | 17 (81) | 9 (50) | 9 (82) |
| *1* | 7 (7.7) | 9 (14) | 7 (17) | 4 (5.7) | 6 (12) | 7 (23) | 3 (14) | 3 (17) | 0 (0) |
| *2* | - | 1 (1.5) | 1 (2.4) | - | 0 (0) | 1 (3.3) | - | 1 (5.6) | 0 (0) |
| *Complication* | 2 (2.2) | 8 (12) | 7 (17) | 1 (1.4) | 3 (6.2) | 5 (17) | 1 (4.8) | 5 (28) | 2 (18) |
| Pneumology grade, n (%) | N=95 | N=70 | N=42 | N=73 | N=52 | N=30 | N=22 | N=18 | N=12 |
| *0* | 46 (48) | 47 (67) | 20 (48) | 32 (44) | 32 (62) | 13 (43) | 14 (64) | 15 (83) | 7 (58) |
| *1* | 38 (40) | 20 (29) | 17 (40) | 31 (42) | 18 (35) | 15 (50) | 7 (32) | 2 (11) | 2 (17) |
| *2* | 3 (3.2) | 1 (1.4) | 4 (9.5) | 2 (2.7) | 0 (0) | 1 (3.3) | 1 (4.5) | 1 (5.6) | 3 (25) |
| *Complication* | 8 (8.4) | 2 (2.9) | 1 (2.4) | 8 (11) | 2 (3.8) | 1 (3.3) | 0 (0) | 0 (0) | 0 (0) |
| Overweight/obesity grade, n (%) | N=94 | N=57 | N=39 | N=72 | N=42 | N=30 | N=22 | N=15 | N=9 |
| *0* | 55 (59) | 32 (56) | 20 (51) | 41 (57) | 25 (60) | 15 (50) | 14 (64) | 7 (47) | 5 (56) |
| *1* | 13 (14) | 4 (7.0) | 5 (13) | 11 (15) | 2 (4.8) | 5 (17) | 2 (9.1) | 2 (13) | 0 (0) |
| *2* | 22 (23) | 17 (30) | 11 (28) | 18 (25) | 13 (31) | 9 (30) | 4 (18) | 4 (27) | 2 (22) |
| *Complication* | 4 (4.3) | 4 (7.0) | 3 (7.7) | 2 (2.8) | 2 (4.8) | 1 (3.3) | 2 (9.1) | 2 (13) | 2 (22) |
| Endocrinology grade, n (%) | N=78 | N=59 | N=39 | N=60 | N=42 | N=28 | N=18 | N=17 | N=11 |
| *0* | 41 (53) | 25 (42) | 21 (54) | 35 (58) | 17 (40) | 15 (54) | 6 (33) | 8 (47) | 6 (55) |
| *1* | 33 (42) | 24 (41) | 14 (36) | 23 (38) | 19 (45) | 10 (36) | 10 (56) | 5 (29) | 4 (36) |
| *2* | 1 (1.3) | - | 1 (2.6) | 1 (1.7) | - | 1 (3.6) | 0 (0) | - | 0 (0) |
| *Complication* | 3 (3.8) | 10 (17) | 3 (7.7) | 1 (1.7) | 6 (14) | 2 (7.1) | 2 (11) | 4 (24) | 1 (9.1) |
| Dermatology grade, n (%) | N=90 | N=70 | N=42 | N=68 | N=52 | N=30 | N=22 | N=27 | N=12 |
| *0* | 56 (62) | 41 (59) | 24 (57) | 47 (69) | 31 (60) | 18 (60) | 9 (41) | 10 (56) | 6 (50) |
| *1* | 22 (24) | 21 (30) | 14 (33) | 13 (19) | 14 (27) | 9 (30) | 9 (41) | 7 (39) | 5 (42) |
| *2* | 1 (1.1) | - | 1 (2.4) | 0 (0) | 7 (13) | 0 (0) | 1 (4.5) | - | 1 (8.3) |
| *Complication* | 11 (12) | 8 (11) | 3 (7.1) | 8 (12) | 31 (60) | 3 (10) | 3 (14) | 10 (56) | 0 (0) |

Supplementary Table 2. Comparison of complications per subpopulation: first, second line and AlloHSCT history

| Characteristic | N | AlloHSCT, N = 12 | First line, N = 66 | Second line, N = 20 | p-value^1^ | q-value^2^ |  |  |  |
| --- | --- | --- | --- | --- | --- | --- | --- | --- | --- |
| Cardiology Visit 1 grading, n (%) | 88 |  |  |  | - | - |  |  |  |
| *0* |  | 3 (30) | 24 (41) | 9 (47) |  |  |  |  |  |
| *1* |  | 5 (50) | 30 (51) | 8 (42) |  |  |  |  |  |
| *2* |  | 0 (0) | 0 (0) | 1 (5.3) |  |  |  |  |  |
| *Complication* |  | 2 (20) | 5 (8.5) | 1 (5.3) |  |  |  |  |  |
| Cardiology Visit 2 grading, n (%) | 63 |  |  |  | 0.86 | >0.99 |  |  |  |
| *0* |  | 3 (38) | 17 (39) | 4 (36) |  |  |  |  |  |
| *1* |  | 5 (62) | 23 (52) | 5 (45) |  |  |  |  |  |
| *2* |  | 0 (0) | 1 (2.3) | 1 (9.1) |  |  |  |  |  |
| *Complication* |  | 0 (0) | 3 (6.8) | 1 (9.1) |  |  |  |  |  |
| Cardiology Visit 3 grading, n (%) | 41 |  |  |  | 0.63 | >0.99 |  |  |  |
| *0* |  | 2 (50) | 11 (37) | 4 (57) |  |  |  |  |  |
| *1* |  | 2 (50) | 12 (40) | 1 (14) |  |  |  |  |  |
| *2* |  | 0 (0) | 1 (3.3) | 1 (14) |  |  |  |  |  |
| *Complication* |  | 0 (0) | 6 (20) | 1 (14) |  |  |  |  |  |
| Nephrology Visit 1 grading, n (%) | 91 |  |  |  | - | - |  |  |  |
| *0* |  | 7 (88) | 56 (89) | 19 (95) |  |  |  |  |  |
| *1* |  | 1 (12) | 5 (7.9) | 1 (5.0) |  |  |  |  |  |
| *Complication* |  | 0 (0) | 2 (3.2) | 0 (0) |  |  |  |  |  |
| Nephrology Visit 2 grading, n (%) | 66 |  |  |  | - | - |  |  |  |
| *0* |  | 10 (91) | 31 (70) | 7 (64) |  |  |  |  |  |
| *1* |  | 1 (9.1) | 6 (14) | 2 (18) |  |  |  |  |  |
| *2* |  | 0 (0) | 1 (2.3) | 0 (0) |  |  |  |  |  |
| *Complication* |  | 0 (0) | 6 (14) | 2 (18) |  |  |  |  |  |
| Nephrology Visit 3 grading, n (%) | 41 |  |  |  | - | - |  |  |  |
| *0* |  | 2 (50) | 18 (60) | 6 (86) |  |  |  |  |  |
| *1* |  | 2 (50) | 5 (17) | 0 (0) |  |  |  |  |  |
| *2* |  | 0 (0) | 1 (3.3) | 0 (0) |  |  |  |  |  |
| *Complication* |  | 0 (0) | 6 (20) | 1 (14) |  |  |  |  |  |
| Pneumology Visit 1 grading, n (%) | 95 |  |  |  | 0.078 | >0.99 |  |  |  |
| *0* |  | 7 (64) | 35 (54) | 4 (21) |  |  |  |  |  |
| *1* |  | 3 (27) | 24 (37) | 11 (58) |  |  |  |  |  |
| *2* |  | 0 (0) | 1 (1.5) | 2 (11) |  |  |  |  |  |
| *Complication* |  | 1 (9.1) | 5 (7.7) | 2 (11) |  |  |  |  |  |
| Pneumology Visit 2 grading, n (%) | 70 |  |  |  | - | - |  |  |  |
| *0* |  | 7 (70) | 31 (67) | 9 (64) |  |  |  |  |  |
| *1* |  | 3 (30) | 12 (26) | 5 (36) |  |  |  |  |  |
| *2* |  | 0 (0) | 1 (2.2) | 0 (0) |  |  |  |  |  |
| *Complication* |  | 0 (0) | 2 (4.3) | 0 (0) |  |  |  |  |  |
| Pneumology Visit 3 grading, n (%) | 42 |  |  |  | - | - |  |  |  |
| *0* |  | 1 (25) | 16 (52) | 3 (43) |  |  |  |  |  |
| *1* |  | 3 (75) | 11 (35) | 3 (43) |  |  |  |  |  |
| *2* |  | 0 (0) | 3 (9.7) | 1 (14) |  |  |  |  |  |
| *Complication* |  | 0 (0) | 1 (3.2) | 0 (0) |  |  |  |  |  |
| Overwheight/obesity Visit 1 grading, n (%) | 94 |  |  |  | - | - |  |  |  |
| *0* |  | 9 (90) | 37 (57) | 9 (47) |  |  |  |  |  |
| *1* |  | 0 (0) | 10 (15) | 3 (16) |  |  |  |  |  |
| *2* |  | 1 (10) | 14 (22) | 7 (37) |  |  |  |  |  |
| *Complication* |  | 0 (0) | 4 (6.2) | 0 (0) |  |  |  |  |  |
| Overwheight/obesity Visit 2 grading, n (%) | 57 |  |  |  | 0.36 | >0.99 |  |  |  |
| *0* |  | 7 (100) | 19 (49) | 6 (55) |  |  |  |  |  |
| *1* |  | 0 (0) | 3 (7.7) | 1 (9.1) |  |  |  |  |  |
| *2* |  | 0 (0) | 14 (36) | 3 (27) |  |  |  |  |  |
| *Complication* |  | 0 (0) | 3 (7.7) | 1 (9.1) |  |  |  |  |  |
| Overwheight/obesity Visit 3 grading, n (%) | 39 |  |  |  | - | - |  |  |  |
| *0* |  | 2 (50) | 15 (52) | 3 (50) |  |  |  |  |  |
| *1* |  | 2 (50) | 1 (3.4) | 2 (33) |  |  |  |  |  |
| *2* |  | 0 (0) | 10 (34) | 1 (17) |  |  |  |  |  |
| *Complication* |  | 0 (0) | 3 (10) | 0 (0) |  |  |  |  |  |
| Endocrinology Visit 1 grading, n (%) | 78 |  |  |  | - | - |  |  |  |
| *0* |  | 6 (67) | 25 (49) | 10 (56) |  |  |  |  |  |
| *1* |  | 3 (33) | 23 (45) | 7 (39) |  |  |  |  |  |
| *2* |  | 0 (0) | 1 (2.0) | 0 (0) |  |  |  |  |  |
| *Complication* |  | 0 (0) | 2 (3.9) | 1 (5.6) |  |  |  |  |  |
| Endocrinology Visit 2 grading, n (%) | 59 |  |  |  | 0.42 | >0.99 |  |  |  |
| *0* |  | 6 (67) | 13 (33) | 6 (55) |  |  |  |  |  |
| *1* |  | 2 (22) | 18 (46) | 4 (36) |  |  |  |  |  |
| *Complication* |  | 1 (11) | 8 (21) | 1 (9.1) |  |  |  |  |  |
| Endocrinology Visit 3 grading, n (%) | 39 |  |  |  | - | - |  |  |  |
| *0* |  | 2 (67) | 15 (50) | 4 (67) |  |  |  |  |  |
| *1* |  | 0 (0) | 13 (43) | 1 (17) |  |  |  |  |  |
| *2* |  | 0 (0) | 1 (3.3) | 0 (0) |  |  |  |  |  |
| *Complication* |  | 1 (33) | 1 (3.3) | 1 (17) |  |  |  |  |  |
| Dermatology Visit 1 grading, n (%) | 90 |  |  |  | - | - |  |  |  |
| *0* |  | 4 (40) | 40 (65) | 12 (67) |  |  |  |  |  |
| *1* |  | 3 (30) | 15 (24) | 4 (22) |  |  |  |  |  |
| *2* |  | 0 (0) | 0 (0) | 1 (5.6) |  |  |  |  |  |
| *Complication* |  | 3 (30) | 7 (11) | 1 (5.6) |  |  |  |  |  |
| Dermatology Visit 2 grading, n (%) | 70 |  |  |  | 0.78 | >0.99 |  |  |  |
| *0* |  | 7 (70) | 25 (54) | 9 (64) | | | |  |  |
| *1* |  | 3 (30) | 15 (33) | 3 (21) | | | |  |  |
| *Complication* |  | 0 (0) | 6 (13) | 2 (14) | | | |  |  |
| Dermatology Visit 3 grading, n (%) | 42 |  |  |  | | | | - | - |
| *0* |  | 3 (75) | 18 (58) | 3 (43) | | | |  |  |
| *1* |  | 1 (25) | 10 (32) | 3 (43) | | | |  |  |
| *2* |  | 0 (0) | 0 (0) | 1 (14) | | | |  |  |
| *Complication* |  | 0 (0) | 3 (9.7) | 0 (0) | | | |  |  |
| ^1^Fisher's exact test | | | | | | |  |  |  |
| ^2^Bonferroni correction for multiple testing | | | | | | |  |  |  |

Supplementary Figure S1


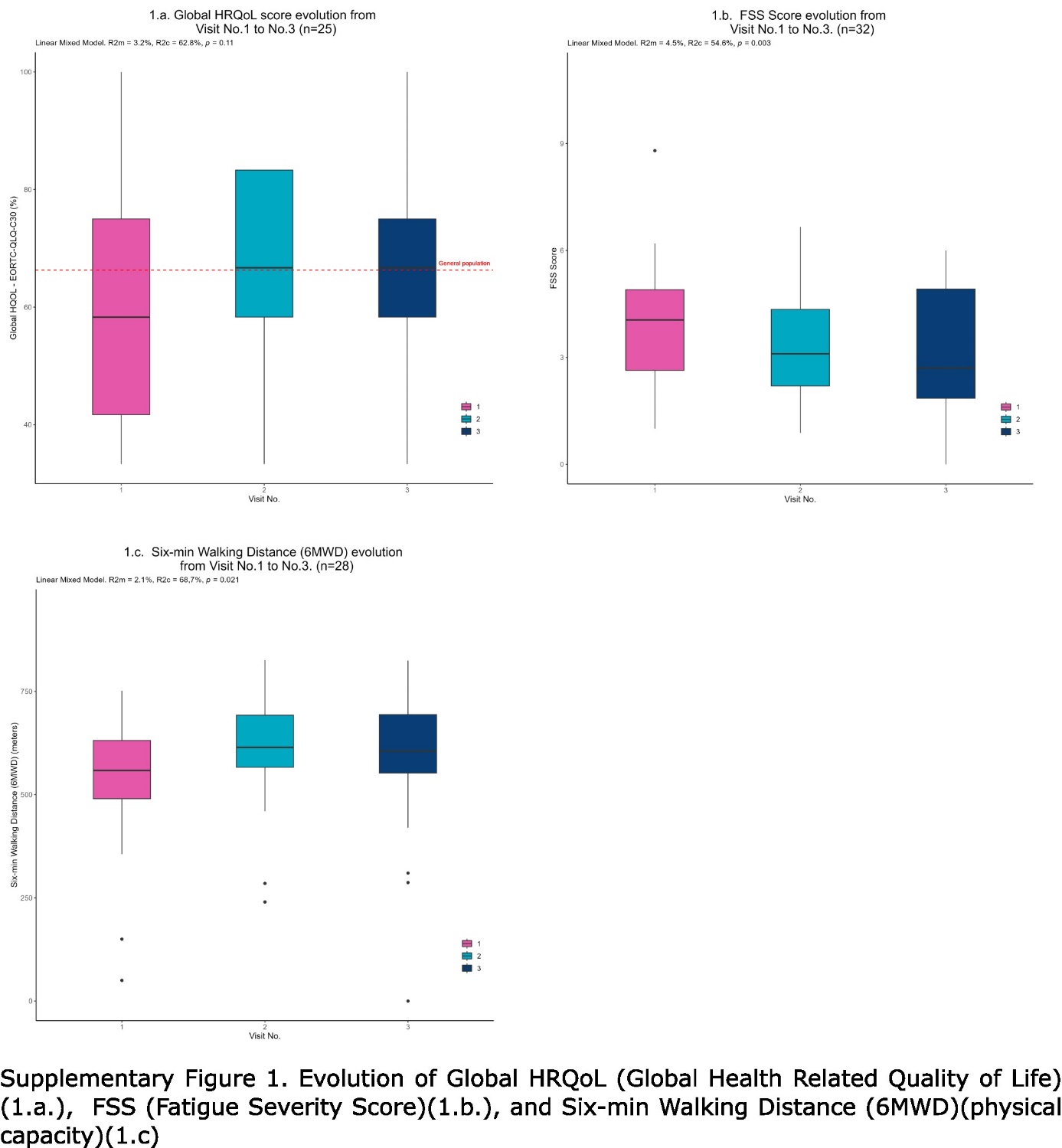

Supplement: Supplementary file 1 [file Supplementary_file_1.docx]
